# Latent Variable Statistical Methods for Longitudinal Studies of Multi-Dimensional Health and Education Data: A Scoping Review

**DOI:** 10.3390/ejihpe15090173

**Published:** 2025-08-28

**Authors:** Meiyang Hong, Jane E. Harding, Gavin T. L. Brown

**Affiliations:** 1 Faculty of Arts and Education, The University of Auckland, Auckland 1142, New Zealand; gt.brown@auckland.ac.nz; 2 Liggins Institute, The University of Auckland, Auckland 1142, New Zealand; j.harding@auckland.ac.nz

**Keywords:** scoping review, methodology, multivariate analysis, latent variable analysis, longitudinal analysis

## Abstract

(1) Background: Most studies including health data have relied on reducing all variables to manifest scores, ignoring the latent nature of variables. Moreover, relying only on manifest variables is a limitation of longitudinal studies where identical measures cannot be collected at each time point. (2) Objective: This scoping review aims to identify latent variable statistical methods for longitudinal studies of multi-dimensional health and educational data investigating early health predictors of long-term educational outcomes and developmental trajectories that lead to better or worse than expected outcomes. (3) Eligibility criteria: We included peer-reviewed health and education journal articles, doctoral theses, and book chapters of longitudinal studies of children under 12 years of age that adopted latent variable, multivariate analysis of three or more waves of data. We only included full-text-available, English-written articles, without restriction on date of publication. (4) Sources of evidence: We searched five databases, Scopus, MEDLINE, PsycINFO, ERIC, and Web of Science, and identified 4836 publications for screening. (5) Results: After title, abstract, and full-text screening, nine studies were included in the review, reporting seven statistical methods. These methods were categorised into two groups—variable-oriented modelling and person-oriented modelling. (6) Conclusions: Variable-oriented modelling methods are useful for determining predictors of long-term educational outcomes. Person-oriented modelling methods are effective in detecting trajectories to better or worse than expected outcomes. (7) Registration: Open Science Framework.

## 1. Introduction

### 1.1. Rationale

In a longitudinal cohort, it is often of interest to examine early predictors and trajectories of outcomes, particularly in health and education domains. The analysis of data waves over a developmental period allows researchers to identify potential risk and protective factors that hinder or contribute to important outcomes. Many studies have investigated the identification of predictors of both health and educational outcomes (Dai et al., 2020; Harwell et al., 2017; S. W. Kim et al., 2019; Kulkarni et al., 2021; Matingwina, 2018; Mayer, 2020), but ignoring the latent nature of data is a limitation of many such studies. Relying only on manifest variables can be problematic in longitudinal studies where identical measures cannot be collected at each time point, which is common among longitudinal studies that look through infancy to adolescence and adulthood. In addition, methods that can detect mid-study changes (for example, environmental shifts) explaining why individuals deviate from trajectories predicted by their early conditions could generate insights not otherwise available. Hence, methods are needed that identify not just which conditions explain outcomes but also which factors contribute to changes in trajectory.

The selection of statistical method may greatly affect the interpretation of results and subsequence policy or clinical suggestions (Silberzahn et al., 2018). A variety of methods have been developed to analyse longitudinal data (Fitzmaurice et al., 2011; Frees, 2004; Hoffman, 2015; Singer & Willett, 2003; Y.-G. Wang et al., 2022). However, uncertainty remains around which method is most appropriate for different research scenarios. This uncertainty typically stems from the nature of data (e.g., manifest vs. latent, categorical vs. continuous), sample size, number of data collection waves, measurement frequency, the nature of missingness, the complexity of outcome variables (e.g., single or multiple), and model assumptions.

A search of systematic or scoping reviews concerning longitudinal statistical methods on Web of Science found most reviews were on specific health issues in medical studies (Cezard et al., 2021; Cosco et al., 2017; Lim et al., 2017; Majewska et al., 2019; Michael et al., 2024; Stanley et al., 2019; Stevens et al., 2021; Timman et al., 2004), but with little focus on educational studies (Hicks & Knollman, 2015). In Cosco et al.’s (2017) review, the most popular latent variable model to group similar participants in longitudinal patterns is growth mixture modelling. Other common latent variable methods reported in the reviews include latent class models (Cezard et al., 2021; Cosco et al., 2017; Lim et al., 2017; Majewska et al., 2019) and group-based modelling (Cezard et al., 2021; Cosco et al., 2017), while less frequently used methods include the Tobit model (Majewska et al., 2019). However, these reviews did not exclusively focus on latent variable methods, and they reported more non-latent than latent variable methods. Among latent variable methods, latent growth curve modelling (Bollen & Curran, 2006) is widely used to examine individual trajectories over time. In addition, the cross-lagged panel model (Lüdtke & Robitzsch, 2021) and the random intercept cross-lagged panel models (Hamaker et al., 2015) have also gained popularity for modelling reciprocal effects using longitudinal data, but they are typically applied in two constructs models. Therefore, we undertook this scoping review of statistical methods that adopt longitudinal, multivariate, latent variable design.

While there are many longitudinal cohort studies worldwide that may benefit from methods described in our review, one example that highlights the need for a comprehensive review of predictor and trajectory analysis methods is the Children with Hypoglycaemia and Their Later Development (CHYLD) study, which explored neonatal and early childhood factors contributing to early schooling outcomes (Shah et al., 2022). This longitudinal cohort study of children born at risk of neonatal hypoglycaemia (low glucose concentrations) includes psychological, environmental, and physiological data in three waves at ages 2, 4.5, and 9–10 years, with educational achievement outcomes at 9–10 years (McKinlay et al., 2015, 2017; Ansell et al., 2017; Shah et al., 2021, 2022). Previous analyses of this dataset applied conventional analysis of variance methods (i.e., “generalized linear models (maximum likelihood) with binomial, Poisson, or normal distributions and relevant link functions, as appropriate, adjusted for prespecified potential confounders (socioeconomic decile, sex, and primary risk factor for neonatal hypoglycaemia).” (Shah et al., 2022, p. 1161). They have relied on manifest scores (e.g., cognitive function, behaviour ratings), ignoring the latent nature of these variables. Modelling these variables as latent variables (e.g., the psychological factor) would enable tests of longitudinal measurement invariance across age given that different measurement instruments/scales were used at different ages in that data. By doing so, researchers may reveal novel insights that previous manifest-only analyses have overlooked.

### 1.2. Objective

The aim of this review was to identify appropriate latent variable statistical methods for studies that have complex and repeated waves of data about the intricate relationships between early health experiences and longer-term educational outcomes. We aimed to offer guidance for researchers facing similar analytical challenges in either disciplinary setting. Three research questions guided this review, as follows:

1. Which latent variable statistical methods have been applied in longitudinal studies of multi-dimensional health and education data?
2. Among those methods found, which methods are appropriate to identify predictors of long-term educational outcomes?
3. Among those methods found, which methods are appropriate to detect trajectories or pathways over time that lead to better or worse than expected educational outcomes?

## 2. Methods

This scoping review was undertaken in accordance with the Preferred Reporting Items for Systematic Reviews and Meta-Analyses extension for Scoping Reviews (PRISMA-ScR) (Tricco et al., 2018). The PRISMA checklist for this scoping review was provided (Supplementary S1). A protocol was prospectively registered with Open Science Framework on 3 May 2023 (https://osf.io/a89rq (accessed on 13 September 2023)).

### 2.1. Eligibility Criteria 

The eligibility criteria are shown in Table 1.

#### 2.1.1. Participants

Studies were eligible if they reported analyses of data from children under 12 years of age. For mixed-age studies, eligibility required that at least 40% of participants were under 12 years, and for multi-wave studies, at least one time point had to include data from participants under 12 years. This age was chosen because in New Zealand, as in many countries, it is the age when many children transition from primary to secondary schooling. Educational influences and opportunities start to undergo significant changes. It is also the time when many children begin to experience significant developmental changes associated with puberty. This threshold was set to minimise potential confounding factors related to adolescence transitions.

#### 2.1.2. Concept

We included multivariate, quantitative, longitudinal design studies. Although linear trends can be identified with two time points (resulting in a saturated model), longitudinal in our review was defined as three or more waves of data, as a minimum of three time points is generally necessary to identify linear trajectories and to evaluate model fit (Bollen & Curran, 2006). Studies could include measures of the same or different variables in multiple waves. Multivariate was defined as at least three outcome variables of interest in the statistical models. In addition, we included only studies with latent variables analysis design, and excluded studies that did not apply latent analysis of manifest variables. We included both methodological articles and application studies but excluded systematic reviews.

#### 2.1.3. Context

We included studies in the fields of health and education that considered human data. Studies reporting analysis of non-human data (such as animals or plants) were excluded. Included studies were peer-reviewed journal articles, doctoral theses, or book chapters with full text available, written in English, with no restrictions on date of publication.

### 2.2. Information Sources and Search

We searched five databases, Scopus, MEDLINE, PsycINFO, ERIC, and Web of Science, from inception to July 2025. The search strategies were developed in consultation with an academic librarian. Key terms used in the search included variations of “statistical method*” (e.g., “statistical analysis”, “statistical model*”, “analytic* approach*”, “analytic* model*”), “multi-variate” (e.g., “multivariate”, “multidimension*”, “multi-construct*”, “multidomain*”), and “longitudinal study” (e.g., “longitudinal studies”, “multi-wave”, “repeated measurement*”). These keywords were applied across five databases, and detailed search strategies are provided in Supplementary S2. All final search results were saved to Zotero (Corporation for Digital Scholarship, 2006/2023) then uploaded into Covidence systematic review software (Veritas Health Innovation, Melbourne, Australia) (available at www.covidence.org (accessed on 12 May 2023)).

### 2.3. Selection of Sources of Evidence

Literature screening was conducted in the following two stages: (1) title and abstract and (2) full texts. The first reviewer (M.H.) carried out the title and abstract screening. Then, at the beginning of full texts screening, two rounds of inter-rater reliability tests were conducted to minimise evidence selection bias. In the first round, three reviewers (M.H., G.B., and J.H.) independently reviewed full texts of the same 30 randomly selected studies. The inter-rater agreement was poor (κ < 0.20) (Fleiss, 1971). To improve the consistency, all reviewers discussed the results and resolved disagreements to reach consensus before the second round. Disagreements were on whether to include mixed-age studies, whether to include studies with simulation data only, whether two-wave studies were considered longitudinal, whether imaging data of humans were considered human data, and whether certain analyses (e.g., cluster analysis) were considered latent variable analysis. In the second round, reviewers worked in pairs (M.H. and G.B., M.H. and J.H.). Each pair independently reviewed 25 randomly selected studies. This resulted in 95% (κ = 0.64) and 96% consensus (κ = 0.65), respectively. Disagreements were on whether doctoral theses were considered grey literature and whether to include review articles. These two rounds of inter-rater reliability tests and discussions increased understanding the subtleties of concept definitions among reviewers and led to refinement of the eligibility criteria. After that, the first reviewer (M.H.) reviewed all remaining studies, with any uncertain cases discussed with the second reviewer (G.B.) to reach a decision.

### 2.4. Data Charting

A data extraction table was jointly developed by all reviewers. Data were extracted from the included studies by the first reviewer and checked by the second reviewer. Data extracted included the context, country, data-related details (sample size, baseline age, number of time points, time intervals, follow-up time, and scale data types), the statistical methods used, and method-related details (handling missing data and software or package).

### 2.5. Synthesis of Results

We classified the identified methods into different groups based on similarities and differences in method assumptions. We also evaluated each statistical method on its suitability for the specific purposes set out in the objectives of review.

## 3. Results

### 3.1. Study Selection

**Figure 1:**
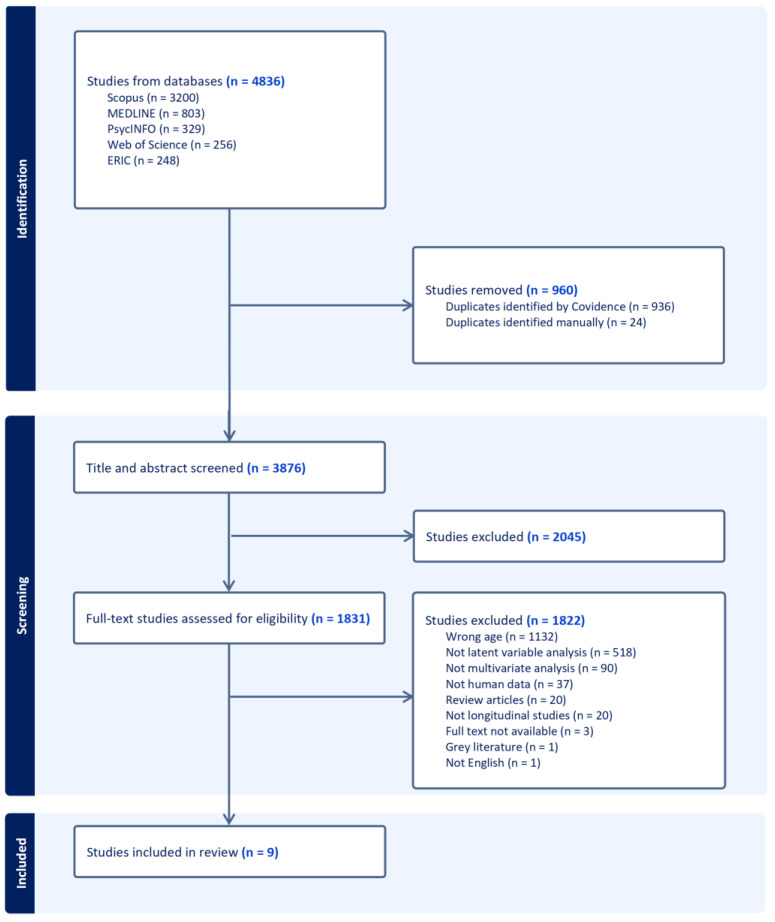
PRISMA flowchart of the selection process.

Figure 1 shows the study selection process. Database searches produced 4836 results, which were uploaded into Covidence. A total of 960 duplicates were removed (936 automatically by Covidence and 24 manually). Of the remaining 3876 studies, 2045 were removed during the title and abstract level screening by the first reviewer (M.H.), leaving 1831 full-text studies to be assessed for eligibility. A further 1822 were removed during full-text screening by three reviewers (M.H., G.B., and J.H.). The most common reasons for exclusion were studies that did not include participants in the right age range (1132) and studies that did not involve latent variables analysis (518). The remaining nine studies were included for further data extraction.

### 3.2. Characteristics of Included Studies

We included nine studies reporting 10 data examples (Table 2). The studies were conducted in diverse countries, with the largest number (i.e., 5 out of 10) in the United States. Sample sizes ranged from 237 to 7507, and follow-up times ranged from 2 to 35 years. Most (6 out of 10) had three or four waves of data. The most common time interval between waves was 1 year (i.e., 6 out of 10), and the most commonly used software for data analysis was Mplus (L. K. Muthén & Muthén, 1998).

### 3.3. Results of Included Studies

From the included studies, we found seven statistical methods (Table 3). Two studies applied latent growth curve modelling (LGM), two growth mixture models (GMM), and two latent transition analysis models (LTA). Each of the other studies adopted a different other method. Three studies identified mean changes in the whole population over time (Isiordia et al., 2017; Ju & Lee, 2018; Liu & Perera, 2023), and six studies investigated trajectories of different subgroups within the population (Ip et al., 2018; Katsantonis & Symonds, 2023; Nagin et al., 2018; Reboussin et al., 1998; Wildeboer et al., 2015; Yarnell et al., 2018).

### 3.4. Synthesis of Results

Based on the assumptions of each method, we grouped the seven methods into two categories—variable-oriented modelling and person-oriented modelling (Table 4). Variable-oriented modelling works on the assumption that people are at the average, while person-oriented modelling looks at the pattens of people within the variables. These two categories indicate that people are not forced to be at the average but allowed to vary within the range. Variable-oriented modelling methods assume population homogeneity and allow researchers to test the influence of variables (Bollen & Curran, 2006), making them suitable for the objective in Research Question 2—identifying predictors of long-term outcome. In contrast, person-oriented modelling methods reflect a person-centred, emergentist worldview drawn from developmental systems theory, assuming population heterogeneity through discrete latent classes, each following its own growth trajectory (B. O. Muthén, 2004). Thus, person-oriented modelling methods are suited to address objective in Research Question 3—detecting trajectories of subpopulation to differential outcomes.

Variable-oriented modelling includes LGM (Ju & Lee, 2018), factor of curves (FOCUS) (Isiordia et al., 2017), and piecewise linear growth curve models (Piecewise LGM) (Liu & Perera, 2023). LGM is a structural equation modelling (SEM) approach that estimates changes over time using two inter-correlated traits (i.e., the starting value or intercept and the change or slope rate) (Brown & Peterson, 2018). LGM can be used to understand group-level change, to explore individual changes, and to identify explanatory variables to predict individual changes (Bollen & Curran, 2006). FOCUS, introduced by McArdle (1988), extends the LGM approach to include higher-order common factors to capture the relations among the lower-order developmental trajectories. Piecewise LGM extends standard LGM by allowing the rate of change (growth/latent slope) to differ across phases separated by one or more knots (timepoints) (Liu & Perera, 2023). Grimm et al. (2016) referred a two-stage piecewise model as a bilinear spline growth model. Using this model, researchers were able to examine joint longitudinal processes. All three methods allow researchers to estimate between-person differences in within-person change in longitudinal studies.

Person-oriented modelling includes GMM, group-based multi-trajectory models (GBMT), hidden Markov models (HMM), and LTA. GMM relaxes the single population assumption of conventional growth modelling to allow for parameter differences across unobserved subpopulations (B. O. Muthén, 2004). GBMT is an extension of univariate group-based trajectory modelling (Nagin et al., 2018), which identifies groups of individuals following similar trajectories over time in terms of a single outcome. GBMT can be used to model multiple related trajectories for more than two outcomes simultaneously (Nagin et al., 2018). HMM is a probabilistic modelling technique that models a system as a Markov process with hidden or unobservable states (latent classes) (Ali & Bouguila, 2022). LTA extends HMM by modelling transitions between latent classes (states) over time. Compared to LGM, which assumes continuous change across an entire time period, LTA uses an autoregressive approach to describe discrete timepoint to timepoint change with transition probabilities (Nylund-Gibson et al., 2023).

## 4. Discussion

### 4.1. Summary

We included 9 studies out of 4836 health and education studies that used latent variable statistical methods to model three or more outcome variables simultaneously, with three or more waves of longitudinal data, conducted with children under 12 years of age. Seven methods were identified from the included studies and classified into two groups: (1) variable-oriented modelling (LGM, FOCUS, and Piecewise LGM) and (2) person-oriented modelling (GMM, LTA, HMM, and GBMT). In general, variable-oriented modelling methods seemed most suitable for determining predictors of long-term outcomes, while person-oriented modelling methods seemed best able to detect trajectories of differential outcomes. We discussed recommendations for analysts with different research purposes below.

### 4.2. Variable-Oriented Modelling: For Researchers Aiming to Find Predictors of Long-Term Outcomes

For analysts looking to understand how one construct influences another construct over time, or to predict in which situation a person will be in the future based on their start values, variable-oriented modelling methods can be used (LGM, FOCUS, and Piecewise LGM) that assume people are close to the average. All three methods are based on the SEM framework, so they share some similarities. They are robust against data limitations and able to handle complex longitudinal data. In contrast to LGM, FOCUS seems to have been used less often in previous studies, but it retains the same ability to model latent factors and structures as LGM (Martinez-Huertas & Ferrer, 2023; McArdle, 1988).

Consistent with SEM, these three methods can model manifest variables as indicators of latent constructs (e.g., standardised test scores and teacher ratings can be aggregated as a latent factor for academic performance). This latent factor approach reduces data dimensionality in a robust manner, making it easier to identify causal paths over time (Bollen, 2020). Hence, they allow for simultaneous modelling of multiple outcome variables and different types of scale data (including nominal, ordinal, and continuous data). Indeed, multiple correlated predictors can be modelled alongside multiple correlated dependencies, along with mediators and moderators. An advantage of Piecewise LGM is that growth at the earlier phase (pre-knot slope) can be specified to predict growth at the later phase (post-knot slope) (Cheong et al., 2003). The piecewise approach in a mediation model is optimal if researchers are interested in finding out the change point event or a developmental milestone (indicated by the estimated knot) in a longitudinal process (Liu & Perera, 2023).

Given the presence of different kinds of data, the SEM framework provides multiple estimators to adjust standard error estimates and test statistics. The use of maximum likelihood estimation allows LGM, FOCUS, and Piecewise LGM to analyse even highly kurtotic data (i.e., up to 7.00) (H.-Y. Kim, 2013). Other estimators exist to handle binary (e.g., asymptotic distribution free) and ordinal (e.g., diagonal weighted least squares) variables. This gives the methods great flexibility.

The methods are designed to model repeated measurements of three or more time points across multiple kinds of time windows; time gaps of more than 2 years are very manageable (Bollen & Curran, 2006; Isiordia et al., 2017; Martinez-Huertas & Ferrer, 2023). This permits tracking changes in data across both small and large time periods. Both LGM and FOCUS are capable of handling relatively small sample sizes of 300–400 (Kline, 2023; Taherian et al., 2021), which are typical in longitudinal cohort studies.

In multiple waves of cohort studies, there is usually an increasing presence of unplanned missing data. The included studies using all three methods used full information maximum likelihood (FIML) to handle missing data. Under a missing at random assumption, both FIML and multiple imputation by chained equations (MICE) are widely used within the SEM framework and can produce unbiased estimates (McArdle & Nesselroade, 2014). It is important to note that multiple imputation are often preferred over FIML (Yucel, 2008), especially when normality assumption is violated or when the model is misspecified (Yuan, 2009). Additionally, Savalei and Bentler (2009) proposed a two-stage approach to handle missing data, which incorporates auxiliary variables to improve estimation. The two-stage approach is more stable than FIML when the sample size is small. In contrast, when person-oriented modelling (e.g., GMM) is used, multiple imputation becomes more complex, and FIML within the mixture modelling framework is commonly used.

In developmental studies, age-appropriate measures are used to estimate a common construct, such as cognitive ability. An important feature of latent factor modelling is that those different indicators can be construed as manifestations of the same underlying construct. Hence, LGM allows different manifest indicators in each data wave to represent the common construct of interest over time. Thus, the robustness of LGM to different indicators at different times is achieved by ensuring indicators are modelled as representing the same underlying construct (McArdle, 2009). Piecewise LGM retains this advantage, while it additionally permits different linear change rates across different phases (Cheong et al., 2003). Consequently, the bias or lack of bias in estimates in LGM depend on factors such as correct model specification, sample size, and estimation methods (Bollen & Curran, 2006; Curran et al., 2010). Although the literature on FOCUS’s robustness to missing data and its ability to produce unbiased estimates is insufficient, its basis in SEM suggests similar performance to that of LGM.

Fortunately, both LGM and FOCUS can be conducted through free software (e.g., R’s lavaan package) (Rosseel, 2012) and Piecewise LGM through R’s OpenMx package (Boker et al., 2025). All three methods also can be performed through commercial software Mplus (L. K. Muthén & Muthén, 1998). These software packages have been widely employed in published studies over the past decade.

### 4.3. Person-Oriented Modelling: For Researchers Aiming to Identify Differential Trajectories

For analysts looking to understand how people are different within a construct over time, or to detect the trajectories of subgroups leading to better or worse than expected outcomes, person-oriented modelling methods can be used (GMM, LTA, HMM, and GBMT). While variable-oriented approaches seek to identify factors contributing to individual differences on the population’s mean trajectory of development, the person-oriented approaches aim at differentiating group membership and understanding how groups respond differently to events that could change a trajectory (Nagin, 2005). Thus, person-oriented approaches provide a valuable framework for identifying factors that lead to unexpected paths. There are likely to be as many as seven possible trajectories over time; those that never change (e.g., always low, middle, or high), those that change consistently in upward or downward direction, and those that move in both directions over time (e.g., up then down or down then up).

With so many possible trajectories, it is likely that overall sample size will have an impact on reliably detecting such trajectories. Thus, a significant concern with person-oriented modelling is the overall sample size being evaluated. This has an impact on the number of trajectory groups being formed and their cell size. In relatively small samples, the approach can create very small trajectory groups as a consequence of random data fluctuations. Consistent with conventional interpretations of the central limit theorem (King et al., 2018), it is generally recommended to exclude a trajectory group smaller than 30 individuals (Nagin et al., 2024). For GMM, the smallest recommended total sample size is 200 (S.-Y. Kim, 2012), although this may sometimes vary depending on the model complexity, number of groups, percentages of missing data, and group separation (S.-Y. Kim, 2014). Similarly, for GMBT, a total sample size of at least 200 is generally sufficient to stabilize the number of trajectory groups identified, ensuring consistent results as sample size increases beyond this threshold (Sampson et al., 2004). The optimal number of groups is the fewest groups needed to represent distinct trajectory groups of the research interest (Nagin et al., 2024). However, no clear consensus exists for the sample size requirement for HMM and LTA (Nylund-Gibson et al., 2023). For LTA, Nylund-Gibson & Choi (2018) suggested a minimum of 300 while Baldwin (2015) suggested 500 or more. Thus, LTA may not be capable of handling relatively small sample sizes of 300–400. Conversely, HMM is applicable to sample sizes in this range (Song et al., 2017), but larger sample size may be required when modelling more latent states and observations to ensure reliable results (Zucchini et al., 2016).

When data are missing at random, all four methods (GMM, GBMT, HMM, and LTA) are generally robust to missing data when techniques such as FIML or expectation maximisation (EM) are applied (Collins & Lanza, 2010; Enders, 2022; Lee & Harring, 2023; Mody et al., 2021; Song et al., 2017). GMM can produce unbiased estimates of standard error, though biased standard error may occur with complex models and small sample size (Bauer & Curran, 2003). GBMT may underestimate standard errors due to the uncertainty in trajectory group assignments when using maximum likelihood estimation alone (Nagin, 2005; Nagin et al., 2018). However, this limitation can be mitigated through bootstrapping to enhance the robustness of standard error estimates (Nagin et al., 2024). For HMM, parameter estimates can be consistent and asymptotically normal under certain regularity conditions, but such conditions are often violated (Zucchini et al., 2016). The sample size has to be very large so that the asymptotic theory of maximum likelihood can apply. Consequently, it is possible that biased standard error estimates occur with a sample size of 300–400.

Regarding flexibility, GBMT can accommodate different outcome measures at different times by using appropriate statistical distributions (e.g., normal, binary or Poisson) (Nagin, 2005; Nagin et al., 2018). However, standardisation or normalisation of outcomes is necessary to ensure robustness when scales differ significantly. HMM can handle repeated measurement with a time interval of more than two years (Song et al., 2017). Its extensions, such as hidden Markov latent variable models, are particularly suitable for longitudinal data involving potentially different outcome measures at different times (Song et al., 2017). LTA, applicable to repeated measurements of three time points (Morin et al., 2021), is similarly robust to different outcome measures, because it does not require consistent measurement across different time points (Nylund-Gibson et al., 2023).

The most widely used software for GMM and LTA includes Mplus (L. K. Muthén & Muthén, 1998) and Latent Gold (Vermunt & Magidson, 2005). Commercial software includes SAS and Stata versions for GBMT (Jones, 2023) and MATLAB-based tools (Wake Forest School of Medicine, 2020). Free software or package options, such as R’s lcmm package for GMM (Proust-Lima et al., 2017), R’s depmixS4 package (Visser & Speekenbrink, 2010) for HMM, and R’s LMest (Bartolucci et al., 2017) and OpenMx (Boker et al., 2025) packages for LTA, offer accessible alternatives for researchers.

### 4.4. Limitations

The first limitation of this review is that it does not include possible AI or machine learning methods applied in health and education. The predictive analytical capabilities of emerging AI technologies would enable identifying potential outcomes and trends not easily captured without such algorithms (Olawade et al., 2023; J. Wang & Li, 2024). Application of AI and machine learning techniques within longitudinal studies may discover new insights into complex health and education trajectories. Exclusion of AI and machine learning may potentially limit the applicability of our findings to contexts that increasingly rely on big data analytics.

Further, potential publication bias exits. By restricting our search to Scopus, MEDLINE, PsycINFO, ERIC, and Web of Science, we may have excluded methods reported in more comprehensive AI based databases (e.g., Dimensions.ai) (Stahlschmidt & Stephen, 2020). Related, there is a limitation because of our choice to restrict publications to English only. Excluding non-English publication could bias our review toward methods popular in English-speaking research communities, where studies using certain statistical methods are more likely to be published and therefore overrepresented in the review. We also excluded studies of children older than 12 years of age, to avoid possible confounding due to the substantial educational and pubertal changes expected after this age. Thus, findings may be less applicable to other age groups.

While we identify seven latent variable methods, the review did not find any articles on the cross-lagged panel model (CLPM) or random-intercept cross-lagged panel model (RI-CLPM) that had three or more constructs. These methods are widespread but are most often specified for bivariate outcome constructs, which explains why few CLPM and RI-CLPM studies met our inclusion criteria. Technically, the cross-lagged panel model can be extended to model three or more variables. However, this increases the number of paths and variables to estimate and often requires a larger sample size. Moreover, Lucas (2023) showed that CLPM often underestimates the actual cross-lagged effects and produces spurious cross-lagged effects when they do not exist, because it fails to account for stable trait-level variance. Increasing model complexity may make the model even more vulnerable. Future research could explore the feasibility of extending the bivariate cross-lagged panel model to one that involves more than two constructs.

In addition, conclusions in our review were drawn from a small number of included studies, with only 1–2 research examples per method. Although we supplemented these findings with a discussion of studies from the broader literature, the small evidence base may limit the generalisability of our findings. Future work could seek to include broader search and identify methods from a larger body of research.

### 4.5. Conclusions

This review identified nine studies employing seven latent variable analytical methods to model three or more outcome variables in longitudinal health and education data involving children under 12 years of age. The results suggest variable-oriented modelling (e.g., LGM) is the most effective for identifying predictors of long-term outcomes and person-oriented modelling (e.g., GMM) for detecting change trajectories. However, without investigating the recent AI and machine learning advancements and more comprehensive databases, we may have overlooked relevant methodologies. Future research should explore these emerging technologies to enhance understanding of child development trajectories in health and education.

## Figures and Tables

**Table 1:** Inclusion and exclusion criteria for studies.

	Inclusion Criteria	Exclusion Criteria
Publication	Peer-reviewed journal articles, doctoral theses, and book chapters with full text available; health and education paper.	Grey literature; full text not available.
Years of publication	No date restrictions.	None.
Language	Accessible in English.	Not accessible in English.
Study design	Longitudinal quantitative studies.	Cross-sectional studies; review articles.
Methodology	Multi-variate analysis; latent variables analysis.	Univariate or bivariate analysis; manifest variable analysis only.
Participants	Humans (<12 years); mixed-age studies: children under 12 > 40% of cohort; multi-wave studies: at least one time point under 12.	Humans (12+ years); mixed-age studies: children under 12 < 40% of cohort; non-human data.

**Table 2:** Characteristics of the included studies (sorted by publication year).

Author(s) (Year)	Context	Country	Baseline Age (Years)	*N*	Follow-Up Period (Years)	Data Waves	Wave Interval (Years)	Scale Data Type	Software or Package
Liu and Perera (2023)	Education	United States	5	400	5.5	9	0.5–1	Continuous	R package OpenMx v 2.21.8
Katsantonis and Symonds (2023)	Health and education	Ireland	3	7507	6	3	2–4	Ordinal; continuous	Mplus v8.7
Yarnell et al. (2018)	Health and education	United States	11.8 (mean)	502	2.5	4	0.5–1	Nominal	Mplus v7
Nagin et al. (2018)	Health	New Zealand; Canada	3; 6	535 1037	35; 11	6–11 5	4–8; 1–4	Ordinal; continuous	Stata SAS platform
Ju and Lee (2018)	Health and education	Korea	Grade 5	2707	4	4	1	Ordinal	Not mentioned
Ip et al. (2018)	Health	United States	2.5–3.5	237	2	3	1	Ordinal	MATLAB-based HMM software
Isiordia et al. (2017)	Education	United States	10.8 (mean)	674	6	4	2	Ordinal	R v3.0.2 lavaan package
Wildeboer et al. (2015)	Education	Netherlands	6.57 (mean)	4781	4.5	3	1.5–3	Ordinal	Mplus v7
Reboussin et al. (1998)	Health and education	United States	8–11	786	5	5	1	Nominal	Fortran

**Table 3:** Results of the included studies (sorted by statistical method).

Author(s) (Year)	Statistical Method	Purpose of the Method
Ju and Lee (2018)	Latent growth curve modelling (LGM)	(1) To explore the developmental trajectories of depression, self-esteem, peer attachment, and child maltreatment across 4 grade years (2) To identify the longitudinal structural relationship among these constructs
Isiordia et al. (2017)	Latent growth curve modelling (LGM)	(1) To identify the correlations among the latent trajectory of parental educational involvement, parental school perception, and children’s academic competence over 4 waves
	Factor of curves (FOCUS)	(2) To test a higher-order common factor among those multiple constructs over time
Liu and Perera (2023)	Piecewise linear growth curve models (Piecewise LGM)	To understand the longitudinal mediational processes among students’ reading, mathematics, and science ability
Katsantonis and Symonds (2023)	Growth mixture models (GMM)	To examine the differential effects of parent styles on population heterogeneity in the joint developmental trajectories of children’s internalising and externalising mental health symptoms
Wildeboer et al. (2015)	Growth mixture models (GMM)	(1) To explore trajectories of parent-reported child aggression across 3 time points (2) To identify the association of child aggression with aggressive behaviour, attention problems, and rule-breaking behaviour
Nagin et al. (2018)	Group-based multi-trajectory modelling (GBMT)	(1) To identify individuals following similar trajectories on multiple disease biomarkers (BMI, forced vital capacity/forced expiratory volume, and arterial blood pressure) (2) To identity individuals following similar trajectories on four behavioural risk factors (childhood physical aggression, violent delinquency, drug use, and sexual partners)
Ip et al. (2018)	Hidden Markov model (HMM)	(1) To identify different feeding styles over time (2) To identify the association between feeding styles, dietary outcomes, and obesity over time
Yarnell et al. (2018)	Latent transition analysis models (LTA)	To identify patterns of multiple risk behaviours (violence, delinquency, substance use) among African American and Hispanic boys over time
Reboussin et al. (1998)	Latent transition analysis models (LTA)	To identify latent health-risk states and the transition of those states over time

**Table 4:** Grouping of statistical methods from included studies.

Group	Group Name	Statistical Method	Research Objective
1	Variable-oriented modelling	Latent growth curve modelling (LGM) Factor of curves (FOCUS) Piecewise linear growth curve models (Piecewise LGM)	Identifying predictors of long-term outcome
2	Person-oriented modelling	Growth mixture models (GMM) Group-based multi-trajectory modelling (GBMT) Hidden Markov model (HMM) Latent transition analysis models (LTA)	Detecting trajectories of subpopulation to differential outcomes

## Data Availability

Not applicable.

## Supplementary Materials

The following supporting information can be downloaded at https://www.mdpi.com/article/10.3390/ejihpe15090173/s1, Supplementary S1: Preferred Reporting Items for Systematic reviews and Meta-Analyses extension for Scoping Reviews (PRISMA-ScR) checklist; Supplementary S2: Search strategies of the five databases.

## Abbreviations

The following abbreviations are used in this manuscript:

AI	artificial intelligence
CHYLD	Children with Hypoglycaemia and Their Later Development
CLPM	cross-lagged panel model
EM	expectation maximisation
FIML	full information maximum likelihood
FOCUS	factor of curves
GMM	growth mixture models
GBMT	group-based multi-trajectory model
HMM	hidden Markov model
LGM	latent growth curve modelling
LTA	latent transition analysis models
MICE	multiple imputation by chained equations
Piecewise LGM	piecewise linear growth curve models
PRISMA-ScR	Preferred Reporting Items for Systematic Reviews and Meta-Analyses extension for Scoping Reviews
RI-CLPM	random-intercept cross-lagged panel model
SEM	structural equation modelling
