# Supplementary material for: Latent Variable Statistical Methods for Longitudinal Studies of Multi-Dimensional Health and Education Data: A Scoping Review
Source: Eur J Investig Health Psychol Educ. 2025 Aug 28;15(9):173. doi: 10.3390/ejihpe15090173 (PMC12468311; doi:10.3390/ejihpe15090173)
Supplement: Supplementary file 1 [file ejihpe-15-00173-s001.zip › ejihpe-3759309-Supplementary S2 updated.pdf]

Supplementary S2: Search strategies of the five databases

1. Scopus search strategy (literature search performed July 28th, 2025)

TITLE-ABS-KEY ("statistical method*" OR "statistical analysis" OR "statistical model*" OR "analytic* approach*" OR "analytic* model*") AND TITLE-ABS-KEY ("multi-variate" OR "multivariate" OR "multidimension*" OR "multi-dimension*" OR "multi-construct*" OR "multiconstruct*" OR "multi-domain*" OR "multidomain*") AND TITLE-ABS-KEY ("longitudinal study" OR "longitudinal studies" OR "longitudinal survey*" OR "multiwave" OR "multi-wave" OR "repeated measurement*" OR "repeatedly measured") AND LANGUAGE (english)

2. MEDLINE and PsycINFO search strategy – Ovid interface (literature search performed July 28th, 2025)

Number	Search terms
1	Longitudinal Studies/
2	(longitudinal study or longitudinal studies or longitudinal survey* or multiwave or multi-wave or repeated measurement* or repeatedly measured).mp.
3	Multi-variate Analysis/
4	(multi-variate or multivariate or multidimension* or multi-dimension* or multi-construct* or multiconstruct* or multi-domain* or multidomain*).mp.
5	models, statistical/
6	(statistical method* or statistical analysis or statistical model* or analytic* approach* or analytic* model*).mp.
7	1 or 2
8	3 or 4
9	5 or 6
10	7 and 8 and 9
11	Limit 10 to English language

Note. mp = title, book title, abstract, original title, name of substance word, subject heading word, floating sub-heading word, keyword heading word, organism supplementary concept word, protocol supplementary concept word, rare disease supplementary concept word, unique identifier, synonyms, population supplementary concept word, anatomy supplementary concept word

3. ERIC search strategy (literature search performed July 28th, 2025)

(longitudinal study OR longitudinal studies OR longitudinal survey* OR multiwave OR multi-wave OR repeated measurement* OR repeatedly measured) AND (multi-variate OR multivariate OR multidimension* OR multi-dimension* OR multi-construct* OR multiconstruct* OR multi-domain* OR multidomain*) AND (statistical method* OR statistical analysis OR statistical model* OR analytic* approach* OR analytic* model*)) AND (la.exact("ENG") AND stype.exact("Scholarly Journals")

4. Web of Science Core Collection search strategy (literature search performed July 28th, 2025)

"statistical method*" OR "statistical analysis" OR "statistical model*" OR "analytic* approach*" OR "analytic* model*" (All Fields) and "multi-variate" OR "multivariate"

OR "multidimension*" OR "multi-dimension*" OR "multi-construct*" OR "multiconstruct*" OR "multi-domain*" OR "multidomain*" (All Fields) and "longitudinal study" OR "longitudinal studies" OR "longitudinal survey*" OR "multiwave" OR "multi-wave" OR "repeated measurement*" OR "repeatedly measured" (All Fields) and English (Language) not Review OR Proceedings Paper (Document Type) not Environmental Sciences OR Plant Sciences OR Agriculture, Dairy & Animal Science OR Agriculture, Multidisciplinary OR Agronomy OR Limnology OR Veterinary Sciences OR Water Resources OR Zoology (Web of Science Categories)
